# Assessing the Effects of Trimethoprim on the Life History Traits of *Anopheles stephensi*

**DOI:** 10.3390/genes17050507

**Published:** 2026-04-25

**Authors:** Mathieu Zamy, Michael Futo, Bianca C. Burini

**Affiliations:** Florida Medical Entomology Laboratory, Institute of Food and Agricultural Sciences, University of Florida, Vero Beach, FL 32962, USA; m.zamy@ufl.edu (M.Z.); mfuto@ufl.edu (M.F.)

**Keywords:** genetic control, *Anopheles stephensi*, population suppression, tet-off system, Trimethoprim (TMP), dihydrofolate reductase, destabilizing domain, antibiotic, life history traits

## Abstract

Background/Objectives: Malaria remains a major global health burden, particularly in sub-Saharan Africa, where the recent invasion and urban expansion of *Anopheles stephensi* are increasing transmission risk in densely populated areas. Conventional vector control strategies, including widespread insecticide application, are progressively losing efficacy due to the rapid spread of resistance. These limitations have accelerated the development of genetic control approaches aimed at either suppressing vector populations or replacing them with genetically modified mosquitoes incapable of transmitting pathogens, with the shared objective of reducing disease transmission. For population suppression strategies, an essential component is a conditional regulatory system that enables precise control of toxic or otherwise deleterious effector proteins. The most widely used platform, the tetracycline-dependent (Tet) system, modulates gene expression in response to tetracycline. However, this system can exhibit leaky expression and variable regulation, which may compromise its reliability and limit its application in certain contexts. The dihydrofolate reductase (DHFR) destabilization domain (DD) system, developed in *Drosophila*, offers an alternative strategy for post-translational control of protein stability. In this system, proteins fused to a destabilization domain are rapidly degraded unless stabilized by the small molecule trimethoprim (TMP), enabling tight and reversible control. In *Drosophila* and prior reports, this system has been associated with relatively low fitness costs, although such effects have not been systematically evaluated in mosquitoes. Before adapting this system for mosquito genetic control, it is therefore essential to assess the impact of TMP exposure on key life-history traits. Methods: Here, we assessed the effects of varying TMP concentrations on mosquito development, survival, and reproductive output. Results: Our results demonstrate that low concentrations of TMP exposure had no detectable effects on immature development, adult survival, or reproductive output under the conditions tested, supporting the implementation of the DHFR-DD system in mosquitoes. Importantly, these effects were dose-dependent, with moderate to high TMP concentrations producing measurable impacts on mosquito fitness. Conclusions: These findings provide a foundational step toward the development of more precise and reliable conditional expression systems for genetic vector control, advancing innovative strategies to mitigate malaria transmission in high-risk regions.

## 1. Introduction

Vector-borne diseases represent a persistent and expanding global health burden, accounting for more than 17% of all infectious diseases and causing over 700,000 deaths annually [1]. These diseases are transmitted by a range of arthropod vectors, including mosquitoes, ticks, fleas, and others, and disproportionately affect populations in low- and middle-income countries, where healthcare infrastructure and sustained vector control programs are often limited [2]. Among the most consequential mosquito-borne diseases are malaria, dengue, Zika, yellow fever, and chikungunya [3]. Malaria remains the most devastating of these infections. In 2023 alone, an estimated 263 million cases and 597,000 deaths were reported worldwide, with the vast majority occurring in sub-Saharan Africa [4]. In this region, transmission is driven primarily by *Anopheles* mosquito species, which are highly competent vectors of *Plasmodium* parasites. Of particular concern is the recent geographic expansion of *An. stephensi*, historically confined to South Asia and the Middle East, into multiple countries in sub-Saharan Africa [5,6]. Unlike many African *Anopheles* species that preferentially exploit rural habitats, *An. stephensi* is well adapted to urban environments, breeding in artificial water containers and thriving in densely populated settings [7]. Its establishment in African cities threatens to reshape malaria epidemiology by increasing transmission risk in urban centers previously considered comparatively low-risk [8].

Conventional malaria control strategies, principally insecticide-based vector control and antimalarial chemotherapy, have substantially reduced disease burden over the past two decades. However, these gains are increasingly threatened. The rapid spread of insecticide resistance in mosquito populations is eroding the effectiveness of long-lasting insecticidal nets and indoor residual spraying [9,10]. Concurrently, the emergence and spread of Plasmodium strains resistant to frontline antimalarial drugs complicate case management and undermine treatment efficacy [6,7,11]. In response to these challenges, genetic control strategies have emerged as promising complementary interventions that are potentially sustainable. Broadly, these approaches fall into two categories: population replacement, in which mosquitoes are engineered to carry traits that reduce or eliminate their capacity to transmit pathogens, and population suppression, which aims to reduce vector abundance through genetic mechanisms [12]. Both strategies aim to disrupt vector-level transmission dynamics, thereby reducing disease incidence in human populations.

Genetic population suppression strategies typically depend on the conditional expression of effector genes that impair mosquito viability, fertility, or overall fitness. By coupling deleterious gene products to tightly regulated expression systems, these approaches enable large-scale laboratory rearing while ensuring that engineered mosquitoes exert their intended impact only after release. A prominent example is the Release of Insects carrying a Dominant Lethal (RIDL) strategy, in which male mosquitoes are engineered to harbor a dominant lethal transgene [13]. In mass-rearing facilities, the lethal phenotype is repressed, allowing colony maintenance. Following release into the field, however, the blocking of the effector gene results in non-viable offspring, leading to progressive population decline [14]. Most RIDL-based systems rely on the tetracycline-controlled transcriptional activation (Tet-off) system to achieve conditional gene regulation. In this framework, tetracycline or its analogs suppress transgene expression during colony maintenance, whereas removal of the antibiotic activates expression in the field. Despite its widespread adoption, the Tet system presents several technical limitations, including basal (leaky) expression in the repressed state [15], variability in transcriptional control across genetic backgrounds or laboratory conditions [16], and in some cases, reduced maximal transgene expression [16]. These constraints can compromise both strain stability and field performance.

These challenges highlight the need for alternative molecular regulatory systems that provide tighter, more predictable, and reversible control of effector gene expression. The development and validation of such systems, including those based on antibiotics or other small-molecule regulators, represent a critical step toward improving genetic population suppression technologies.

Conditional gene-regulation systems in mosquitoes and other insects have employed antibiotics or small molecules as external regulators to achieve temporal, reversible control of gene expression [16,17,18,19,20,21]. Studies using antibiotic-mediated microbiota perturbation have demonstrated that small molecules can also exert broad, unintended physiological effects on mosquito physiology or pathogen development, underscoring the multiple pathways through which chemical exposures can shape mosquito biology [22,23,24,25]. If antibiotics are used as the molecular switch in conditional gene-regulation systems, their effects on mosquito physiology must be carefully evaluated. This assessment is essential to determine the feasibility of deploying such systems in genetic control strategies.

In this context, the *Escherichia coli* dihydrofolate reductase–based destabilizing domain (ecDHFR-DD) system offers a post-translational approach to conditional gene regulation that has been well validated in *Drosophila melanogaster*. In this system, target proteins are fused to a destabilizing domain derived from ecDHFR, which causes the fusion protein to be degraded in the absence of ligand. The introduction of Trimethoprim (TMP), a synthetic antibiotic that inhibits bacterial DHFR, stabilizes the fusion protein by specifically binding to it and preventing its proteasomal degradation. This interaction enables reversible, dose-dependent control of protein accumulation. Using this approach, Sethi and Wang (2017) [16] demonstrated precise temporal control of protein expression across multiple tissues in *Drosophila*, with rapid induction upon TMP administration, efficient depletion upon ligand withdrawal, minimal basal expression in the off state, and limited detectable toxicity. Together, these results highlight the ecDHFR-DD system as a versatile post-translational regulatory system and suggest that TMP-mediated protein stabilization may provide a promising alternative to transcription-based inducible systems for achieving conditional gene control in insects.

This study investigates the feasibility of using small-molecule antibiotics as regulatory agents in adapting the DHFR-DD system in *An. stephensi*. Specifically, we evaluated the effects of TMP on key life-history traits, including development, survival, and reproductive fitness. Understanding how TMP and similar compounds interact with mosquito physiology is essential for assessing their suitability in future genetic control interventions.

## 2. Materials and Methods

### 2.1. Mosquito Rearing

The *An. stephensi* STE2 strain (obtained from BEI Resources, NIAID, NIH, Manassas, VA, USA MRA-128) was maintained under standard insectary conditions: 28 ± 2 °C, 75 ± 5% humidity, and a 12-h light/12-h dark cycle in an Arthropod Containment Level 2 insectary chamber at the Florida Medical Entomology Laboratory, University of Florida. The adult colony was nourished with a 10% sucrose solution *ad libitum*. Blood meals were offered with defibrinated bovine blood (HemoStat Laboratories, Dixon, CA, USA) using an artificial feeding method for experiments and maintenance. Larval stages were maintained in dechlorinated water and fed Tetramin Tropical Flakes (Blacksburg, VA, USA).

### 2.2. TMP Regimen

**Figure 1:**
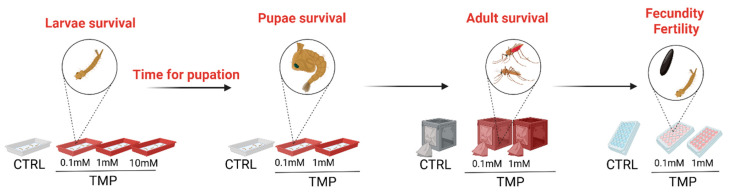
Schematic overview of the experimental design used to evaluate the effects of trimethoprim (TMP) on the life-history traits of *Anopheles stephensi*. Diagrammatic representation of the TMP exposure regimen and downstream phenotypic assessments. Larvae were reared in pans containing defined concentrations of TMP (0.1–10 mM), administered throughout the developmental period. For each concentration, key life-history parameters were quantified across developmental stages. These included: larval survival, time to pupation, pupal survival, adult survival, and adult reproductive output, measured as fecundity (number of eggs laid per female) and fertility (egg hatch rate).

To determine the impact of TMP exposure on mosquito development and overall fitness, we conducted a dose–response assessment across the immature and adult life stages of *An. stephensi* (Figure 1). Newly hatched larvae were transferred to 500 mL water containers supplemented with four TMP concentrations: 0 mM (untreated control), 0.1 mM, 1 mM, and 10 mM (Figure 1). The TMP (cat. no. sc-203302A) used was purchased from Santa Cruz Biotechnology (Dallas, TX, USA). The selected concentrations were informed by previously reported effective doses in *Drosophila*, including the destabilization-domain characterization described by Sethi et al. [1], and were chosen to span a range of exposure levels from low to high. Each container with larvae was provided with fish food (Tetramin), which was kept continuously available throughout the experiment to ensure constant larval feeding. Larval rearing water was replaced every 4 days to maintain water quality and prevent the buildup of food residues, and TMP was replenished to the nominal concentration at each water change to maintain the intended treatment levels. TMP treatment was maintained until adult emergence. Following eclosion, adults from each treatment group continued to receive TMP at the respective concentration administered in a 10% sucrose solution, thereby maintaining continuous exposure during the adult stage for subsequent survival and reproductive assessments (Figure 1). The sucrose–TMP solution was freshly prepared and replaced every two days to maintain consistent exposure. Mosquitoes had continuous access to the solution within each cage, although individual consumption was not directly measured.

Trimethoprim is fully soluble only in DMSO. However, initial tests revealed that even minimal DMSO exposure caused complete mortality of *An. stephensi* larvae within 72 h (Supplemental Material Table S1), indicating that DMSO is highly toxic even at very low concentrations and highlighting the need to carefully optimize TMP dosing in mosquito assays. To avoid solvent-related toxicity, TMP was prepared in sterile distilled water as a heterogeneous suspension at a nominal concentration of 100 mM. Because TMP does not fully dissolve in water, the suspension was vortexed vigorously for 15 s immediately prior to dilution into larval containers to ensure uniform dispersion of the compound. Aliquots from these freshly vortexed suspensions were then diluted to nominal final concentrations of 0.1 mM, 1 mM, and 10 mM. Control larvae received an equivalent volume of water without TMP. Although these concentrations represent nominal exposure levels rather than analytically verified dissolved TMP, all treatments were prepared using identical procedures immediately prior to use, allowing consistent, reproducible comparisons of dose-dependent biological effects.

### 2.3. Larval and Pupal Survival and Pupation Time

Newly hatched *An. stephensi* larvae were transferred to polypropylene rearing containers (2.5″ depth; 11.5″ × 17.5″) containing 500 mL of water supplemented with TMP (0, 0.1, 1, or 10 mM). Each container was seeded with 65 first-instar larvae to standardize density across treatments, and all groups received identical feeding regimens in both quantity and timing to eliminate nutritional variability as a confounding factor. Larvae and pupae were monitored daily from hatching through pupation, and mortality was recorded at each observation point. Pupation time was determined by recording the number of days required for larvae to reach the pupal stage. Pupae were collected daily and transferred to adult emergence cages according to the day of pupation.

#### Adult Survival

To evaluate adult survival, *An. stephensi* individuals derived from each TMP treatment group were maintained under continuous exposure to their respective concentrations. Following emergence, adults were transferred to rearing cages and provided *ad libitum* access to a 10% sucrose solution supplemented with the same TMP concentration administered during the larval stage (0, 0.1, or 1 mM), thereby ensuring uninterrupted treatment across life stages. The 10 mM treatment was excluded from the adult survival analyses because it resulted in complete larval mortality, and no adults emerged.

### 2.4. Fecundity and Fertility

Adult females, reared as larvae in TMP concentrations of 0 (control), 0.1 mM, or 1 mM, were maintained on 10% sucrose solutions containing the same TMP concentrations until they were artificially blood-fed. Fully engorged females were placed individually in tubes with water for oviposition 72 h post-blood feeding. After 3–4 days, images of the eggs were captured for counting using ImageJ (version 1.53t, NIH, Bethesda, MD, USA). Following hatching, larvae were transferred to containers, fed, and allowed to develop for 5 days before additional images were taken for counting in ImageJ. Fecundity was defined as the total number of eggs laid per female, and fertility was defined as the proportion of eggs that successfully hatched, calculated by dividing the number of larvae that emerged by the total number of eggs laid per female.

### 2.5. Statistical Analyses

All experiments included three independent biological replicates, each derived from eggs laid by a distinct cohort of females. While the data from all replicates were combined for analysis, each replicate represents an independent biological source, ensuring that the dataset reflects biological variability. Statistical tests were applied to the pooled dataset. Differences in survival among larvae and adults exposed to TMP (Control, 0.1 mM, and 1 mM) were evaluated using the log-rank (Mantel–Cox) test. Pairwise comparisons among survival curves were adjusted for multiple testing using the Bonferroni correction. The 10 mM group was excluded from adult survival analyses because no individuals survived to adulthood. For pupation time, fecundity, and fertility, Normality was evaluated using D’Agostino–Pearson, Anderson–Darling, Shapiro–Wilk, and Kolmogorov–Smirnov tests. Multiple tests were applied because each has distinct sensitivities, and concordance among tests was used to guide interpretation. If tests indicated a significant deviation from normality (*p* < 0.05), nonparametric methods were used; otherwise, parametric methods were used. A *p* value < 0.05 was considered statistically significant. Significance levels are indicated as follows: ns: *p* > 0.05; *: *p* ≤ 0.05; **: *p* ≤ 0.01; ***: *p* ≤ 0.001; and ****: *p* ≤ 0.0001. All statistical analyses and graphical visualizations were performed using GraphPad Prism version 10.3.1 (GraphPad Software, San Diego, CA, USA).

## 3. Results

Aquatic development in *Anopheles stephensi* exhibits dose-dependent sensitivity to TMP with no detectable effects on immature development at low doses.

**Figure 2:**
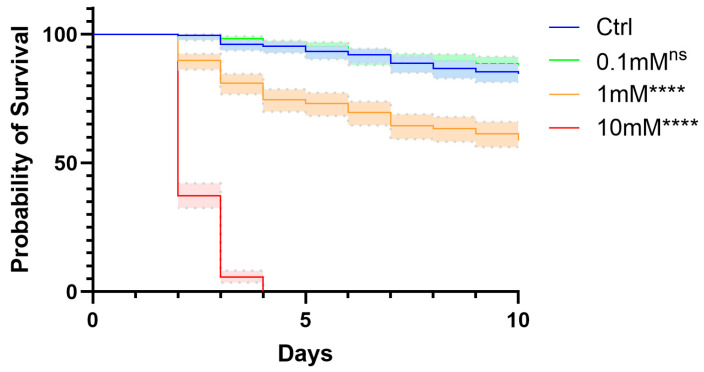
Survival of *Anopheles stephensi* larvae exposed to trimethoprim. Survival curves of larvae reared in water containing 0 mM (control), 0.1 mM, 1 mM, or 10 mM TMP over a 10-day observation period. A total of 195 newly hatched larvae were analyzed per treatment group (65 per replicate; three independent biological replicates). Statistical significance was assessed using the Log-rank test, with *p* values adjusted for multiple comparisons using the Bonferroni method (**** *p* ≤ 0.0001, ns: not significant).

To evaluate the impact of TMP exposure on key life history traits of *An. stephensi*, we first assessed aquatic stages at concentrations of 0.1 mM, 1 mM, and 10 mM TMP, with a control group maintained without TMP. Newly hatched larvae were placed in larval trays containing TMP at the designated concentrations. Larvae were observed every 24 h, and dead individuals were counted and removed. A total of 195 larvae were evaluated per TMP treatment. Exposure to 0.1 mM TMP had no discernible effect on larval survival. In contrast, treatment with 1 mM TMP markedly reduced larval survival, with only 58.9% of individuals reaching pupation. The highest concentration tested, 10 mM TMP, had a severe effect, resulting in complete mortality within four days (Figure 2). These results demonstrate a clear, dose-dependent impact of TMP on *An. stephensi* larval survival and highlight that the lowest dose tested (0.1 mM) was well tolerated under our conditions.

**Figure 3:**
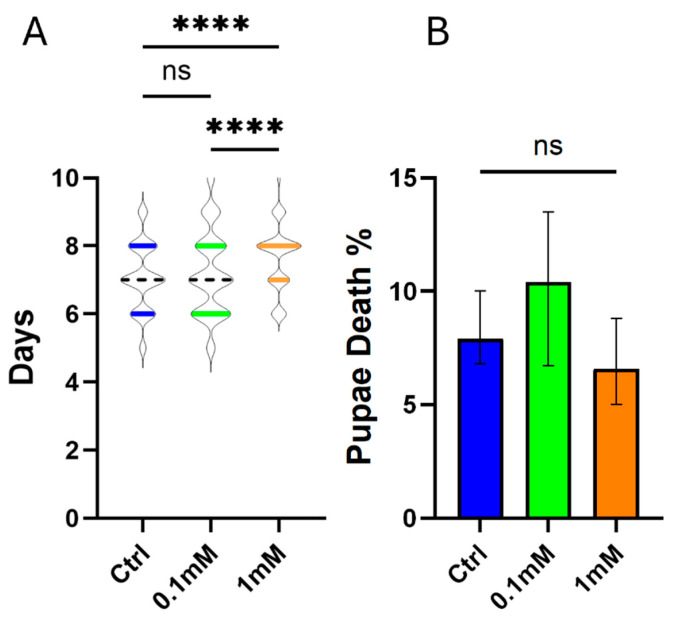
Effect of TMP exposure on pupation time and pupal mortality in *Anopheles stephensi*. (**A**) Time to pupation for larvae exposed to 0 mM (control), 0.1 mM, and 1 mM TMP. Pupation time was recorded daily from hatching until pupation. Data are presented as median ± interquartile range. (**B**) Pupal mortality for the same treatment groups was recorded daily for all pupae until adult emergence. Data are presented as median ± interquartile range. Statistical comparisons among groups in both graphs were performed using the Kruskal–Wallis test, followed by Dunn’s multiple comparisons test. (**** *p* ≤ 0.0001, ns: not significant).

To further evaluate the impact of TMP on aquatic stages, pupal development was assessed across treatment groups (0 mM, 0.1 mM, 1 mM, and 10 mM TMP). Larvae were monitored daily to record the onset and progression of pupation. In the control and 0.1 mM TMP groups, pupation occurred within approximately 7 days, consistent with the standard developmental timeframe observed under our colony maintenance conditions (Figure 3A). Larvae exposed to 1 mM TMP reached pupation after approximately 8 days, representing a statistically significant delay compared to the control and 0.1 mM groups, suggesting that TMP at this concentration imposes mild developmental stress (Figure 3A). No pupation was observed in the 10 mM group due to complete larval mortality, as mentioned previously.

In addition to pupation timing, pupal mortality was monitored across the surviving groups. No significant differences were observed between the control, 0.1 mM, and 1 mM TMP treatments, indicating that TMP exposure at concentrations compatible with larval survival does not adversely affect pupal viability (Figure 3B). These results collectively suggest that TMP has no effect on pupal development at 0.1 mM. At 1 mM, there is only a minor delay of one day, and neither dose affects mortality.

TMP Exposure Does Not Reduce Adult Survival in *Anopheles stephensi* at low doses.

**Figure 4:**
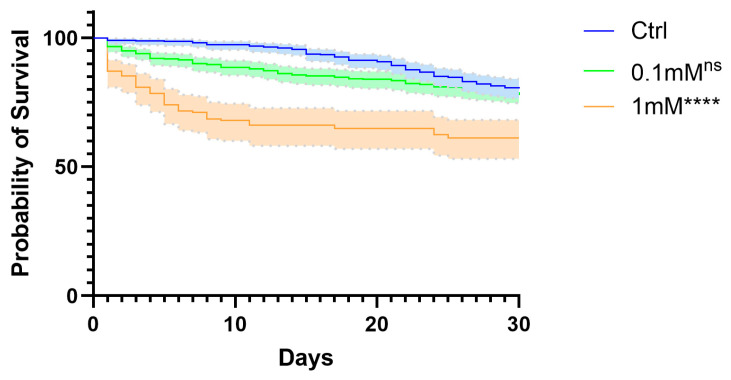
Survival of *Anopheles stephensi* adults exposed to trimethoprim. Survival curves of adult *Anopheles stephensi* over 30 days following exposure to 10% sucrose containing 0 mM (control), 0.1 mM, or 1 mM TMP. Adults were derived from larvae reared under identical conditions. Experiments were conducted in triplicate, with n per group as follows: control, 330; 0.1 mM, 315; 1 mM, 162. Survival was recorded daily. Statistical differences were assessed using the Log-rank test (Mantel–Cox) with Bonferroni correction (**** *p* ≤ 0.0001; ns, not significant), and dashed areas represent the 95% confidence interval (CI).

To assess adult survivorship following TMP exposure, newly emerged adults from each treatment group were monitored daily for 30 days, and mortality was recorded throughout the observation period. Adults from the control group exhibited the highest survival over time. Exposure to 0.1 mM TMP resulted in a modest reduction in survival, which was not statistically significant after Bonferroni correction. In contrast, exposure to 1 mM TMP resulted in a pronounced decrease in longevity compared with controls (Figure 4).

**Figure 5:**
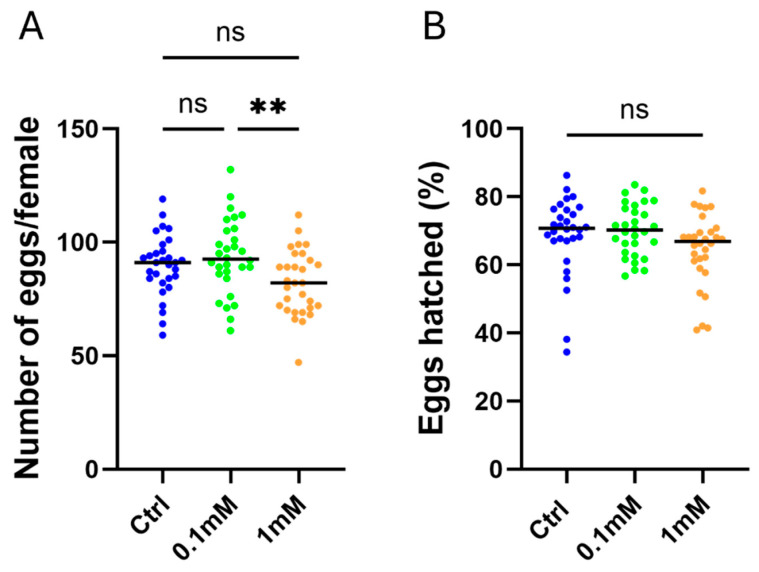
Effects of TMP exposure on reproductive output in *Anopheles stephensi*. (**A**) Fecundity, expressed as the number of eggs laid per female, for adults derived from the 0 mM (control), 0.1 mM, and 1 mM TMP groups. Each point represents an individual female following an artificial blood meal: *n* = 30 (0 mM), 30 (0.1 mM), 30 (1 mM). Egg production was recorded over a two-day oviposition period starting three days post–blood feeding. (**B**) Fertility, expressed as the percentage of eggs hatched per female, for the same treatment groups. Experiments were performed with three biological replicates. Statistical analyses were conducted using the Kruskal–Wallis test, followed by Dunn’s post hoc multiple comparisons test. (** *p* ≤ 0.01; ns: not significant).

To assess the effects of TMP exposure on female reproductive performance, both fecundity and fertility were measured across treatment groups. Adult females from each treatment group were blood-fed and allowed to oviposit individually. The total number of eggs laid per female was recorded to quantify fecundity. Females from the control, 0.1 mM, and 1 mM TMP groups successfully oviposited following blood feeding. While fecundity in the 0.1 mM group was comparable to that in the control, females exposed to 1 mM TMP exhibited a mild but statistically significant reduction in the number of eggs laid per female relative to both the control and 0.1 mM groups (Figure 5A).

To assess fertility, eggs from individual females were monitored for larval hatching, and hatch rate was calculated as the proportion of eggs that successfully produced larvae. No significant differences in hatch rate were observed among the control, 0.1 mM, and 1 mM TMP groups (Figure 5B), indicating that TMP exposure at concentrations up to 1 mM does not impair egg viability despite the modest reduction in egg production at the higher dose.

## 4. Discussion

This study evaluated the tolerance of *An. stephensi* to TMP across a range of concentrations to assess its suitability as a regulatory molecule for the DD-ecDHFR gene expression system [17,18]. Our findings reveal concentration-dependent effects of TMP on mosquito survival and development, while identifying a low-dose window that causes no significant biological disruption.

Larval survival was strongly influenced by TMP concentration. At 0.1 mM, TMP had negligible effects on larval survivorship compared with controls, indicating that low-dose exposure does not cause acute developmental toxicity. In contrast, survival decreased at 1 mM and was complete at 10 mM, demonstrating a clear dose-dependent threshold for toxicity. Disruption of the larval microbiota through antibiotic treatment can significantly impair development and survivorship, underscoring the indispensable role of gut microbes in mosquito physiology [19,20]. Prior studies have shown that antibiotic exposure during larval stages can compromise growth and survival through perturbation of microbial communities rather than direct cytotoxic effects [21,22,23]. These findings support the interpretation that higher TMP concentrations may disrupt microbiome-associated processes essential for larval nutrition and metabolic homeostasis [24,25]. Notably, sublethal concentrations in our study did not produce rapid mortality or overt developmental arrest, suggesting that TMP toxicity at 1 mM likely reflects physiological stress rather than direct tissue damage.

Trimethoprim exposure also influenced developmental timing in a concentration-dependent manner. Larvae reared at 0.1 mM pupated within the expected timeframe, comparable to controls. However, individuals exposed to 1 mM TMP exhibited a delay of approximately 24 h in pupation, indicating interference with developmental progression at higher concentrations. Developmental delays of similar magnitude have been reported in mosquitoes exposed to microbiota disruption, nutritional stress, or metabolic inhibitors [19,22,26], supporting the interpretation that delayed pupation reflects generalized physiological stress rather than system-specific toxicity. TMP-dependent developmental slowing has likewise been observed in *D. melanogaster* during the implementation of post-translational regulatory systems, where increasing TMP concentrations extended larval development relative to untreated controls [1]. While TMP was administered at defined nominal concentrations in the aquatic stages, its stability in aqueous conditions was not directly assessed, and concentrations were not experimentally verified over time. Therefore, although continuous exposure was intended, potential fluctuations in TMP levels during the experiment cannot be excluded.

A previous study demonstrated small-molecule regulation of gene drive activity using a Cas9 protein fused to the DHFR destabilization domain, where Cas9 stability and activity were controlled by TMP [27]. In that system, TMP concentrations of ~0.08 mM were required for effective activation, though such levels caused developmental delays of 5–10 days in *Drosophila*. Because our study uses the same TMP-based destabilization-domain framework, these findings provide useful context for interpreting the tolerance window observed in *An. stephensi*. Notably, the TMP concentration required for DD-Cas9 activation in *Drosophila* is comparable to the 0.1 mM TMP concentration identified here as biologically tolerable. These observations highlight the potential to achieve effective DD-based regulation while minimizing fitness impacts, suggesting that TMP concentrations sufficient to stabilize DD-fused proteins can be achieved in mosquitoes.

It is also important to consider the expression context. In *Drosophila*, the ecDHFR-DD system was tested using the GAL4-UAS overexpression system [16], which produces high transgene levels. Because destabilization-domain systems rely on the balance between protein synthesis and degradation, stabilization dynamics can vary with expression strength. Mosquito constructs for vector control typically involve single genomic insertions with moderate promoters, resulting in lower, more tightly regulated expression. Therefore, while *Drosophila* studies provide proof of principle that TMP can regulate DD-fused proteins in vivo, additional studies in mosquito systems are needed to determine how effectively this strategy functions under vector-control-relevant expression conditions.

Adult longevity was not reduced at 0.1 mM TMP relative to controls, indicating that low-level TMP exposure does not impose a detectable survival cost. In contrast, a higher concentration (1 mM) did reduce adult lifespan, suggesting that chronic exposure at elevated levels may generate cumulative physiological stress that manifests later in life. Similar reductions in adult longevity following chronic antibiotic exposure have been reported in mosquitoes, where sustained physiological stress and altered metabolic homeostasis are proposed as underlying mechanisms [23]. Decreased lifespan in these contexts has been associated with prolonged stress responses and shifts in energy allocation, even in the absence of overt reproductive impairment [22,28]. These parallels support the hypothesis that TMP may subtly disrupt metabolic or immune balance, potentially through microbiome-mediated pathways. The mosquito microbiome is increasingly recognized as a key determinant of host nutrition, metabolism, immune function, and detoxification capacity [24,29]. Alterations in microbial composition have been shown to influence survival outcomes in a life-stage- and exposure-dependent manner [28,30]. Although microbiome composition was not directly assessed in this study, the lack of acute mortality at sublethal concentrations, combined with preserved reproductive capacity, is more consistent with indirect microbiome-mediated effects than with direct cytotoxicity. Confirmation of this mechanism will require targeted microbial and metabolic analyses in future work.

In contrast to the survival and developmental effects observed at higher TMP concentrations, reproductive output was comparatively resilient. Neither treatment group differed significantly from controls in fecundity; however, a significant difference was detected between the 0.1 mM and 1 mM groups. Fertility remained unaffected across all tested concentrations. Preservation of egg hatch rates aligns with prior *Anopheles* studies showing that antibiotic exposure had minimal impact on fertility [21,22]. In *An. stephensi*, antibiotic-mediated gut sterilization via tetracycline delivered in glucose solution significantly reduced oviposition while leaving egg hatch largely unaffected [28], demonstrating that fecundity and fertility can respond independently to microbiome perturbation. The dissociation observed here, a modest variation in egg production at higher TMP levels with stable fertility, suggests that embryogenesis and egg viability remain robust even when subtle physiological stress is present. Importantly, the absence of significant reproductive impairment at 0.1 mM TMP supports the suitability of this concentration for DD-ecDHFR-based gene regulation in *An. stephensi*.

While this study provides important insights into TMP tolerance, it also defines clear avenues for further refinement. In addition to the life-history traits evaluated here, future work incorporating metrics such as adult body size, mating competitiveness, flight performance, extended longevity, and vector competence will enable a more comprehensive assessment of fitness. A key next step will be the direct characterization of microbiome dynamics under TMP exposure. Although our findings are consistent with microbiome-mediated effects, we did not quantify microbial composition or abundance. Integrating approaches such as 16S rRNA gene sequencing to resolve community structure, alongside quantitative assays (e.g., qPCR) to measure bacterial load, will be essential to determine whether the observed phenotypes are associated with shifts in microbial diversity or density. Complementary analyses of metabolic profiles and immune gene expression will further clarify whether altered energy homeostasis or stress-response pathways contribute to the reduced adult survival observed here. In this study, TMP exposure was continuously applied from the larval stage through adulthood to provide a comprehensive assessment of physiological tolerance across the full life cycle and detect potential cumulative effects. While this design is well-suited for feasibility evaluation, future studies incorporating stage-specific exposure regimes (e.g., larval-only or adult-only) will more closely align with operational deployment scenarios and help refine the practical application of the DD-ecDHFR system. The TMP concentrations used here were initially guided by destabilization-domain studies in *Drosophila*, but direct translation to mosquitoes is limited by differences in exposure route, physiology, and detoxification capacity. The present study evaluates relatively broad intervals (0.1–10 mM), with no detectable effects at 0.1 mM but substantial impacts at 1 and 10 mM, indicating that the functional threshold likely lies within the 0.1 to 1 mM range. Future work using finer concentration gradients will be necessary to determine the minimal effective dose for protein stabilization while minimizing fitness costs.

Collectively, these findings delineate a functional window for TMP application in *An. stephensi*. Low-dose TMP (0.1 mM) is largely developmentally and reproductively benign, whereas higher concentrations (1 and 10 mM) impose measurable survival and developmental costs, indicating that the functional threshold for effective DD-ecDHFR stabilization likely lies between these concentrations. These results highlight the importance of considering microbiome-associated effects, physiological stress, and cumulative life-stage impacts when implementing antibiotic-regulated genetic tools. Importantly, with careful optimization of dosing and exposure regime, TMP-regulated DD-ecDHFR systems can be deployed effectively in mosquitoes while minimizing unintended biological impacts. Moreover, our findings encourage the use of this system as a versatile tool in mosquito research, enabling controlled studies of gene function, physiology, and life-history traits and supporting future applications in operationally relevant applications.

## Data Availability

The original contributions presented in this study are included in the article Further inquiries can be directed to the corresponding author.

## Supplementary Materials

The following supporting information can be downloaded at: https://www.mdpi.com/article/10.3390/genes17050507/s1, Table S1: Larval mortality following exposure to TMP and DMSO.
