# Supplementary material for: Assessing the Effects of Trimethoprim on the Life History Traits of Anopheles stephensi
Source: Genes (Basel). 2026 Apr 25;17(5):507. doi: 10.3390/genes17050507 (PMC13205967; doi:10.3390/genes17050507)
Supplement: Supplementary file 1 [file genes-17-00507-s001.zip › genes-4196390-supplementary.pdf]

Supplementary Material

1. **Supplementary Methods**

Mosquito Rearing and Experimental Setup

Larvae of *Anopheles stephensi* were reared under standard insectary conditions. For the experiment, groups of 63–65 larvae per treatment were transferred to plastic pans containing the respective treatment solutions, and Tetramin fish food was added to each pan.

Treatment Conditions

Larvae were exposed to trimethoprim (TMP) dissolved in DMSO at concentrations of 0.1 mM, 1 mM, and 10 mM. Parallel treatments were conducted using the same TMP concentrations dissolved in DMSO supplemented with 10% fetal bovine serum (FBS). To assess solvent toxicity, additional control groups were exposed to DMSO diluted in water at final concentrations of 0.1%, 1%, and 10%. A control group maintained in water without DMSO or TMP was included for comparison.

Larval mortality was recorded at 24, 48, and 72 hours post-exposure. Larvae were considered dead if they showed no movement following gentle mechanical stimulation. The number of surviving larvae was recorded at the final time point for each treatment.

2. **Supplementary Results**

Exposure to DMSO resulted in high levels of toxicity in *An. stephensi* larvae across all tested conditions. In treatments where TMP was dissolved in DMSO, with or without 10% FBS, complete mortality was observed within 48 hours. Mortality was evident at 24 hours and reached 100% by 48 hours in all cases (Supplementary Table S1A).

In DMSO-only treatments prepared in water, toxicity was concentration- and time-dependent. At concentrations of 1% and 10%, DMSO caused complete larval mortality within 24 hours. At 0.1% DMSO, mortality was delayed, with partial mortality observed at 24 and 48 hours, and complete mortality was reached at 72 hours. No larvae survived beyond 72 hours in any treatment containing DMSO. In contrast, all larvae in the untreated control group remained alive throughout the experiment (Supplementary Table S1B).

These results indicate that DMSO alone is sufficient to induce complete mortality in *An. stephensi* larvae under the tested conditions, and is therefore incompatible as a solvent for dissolving TMP in experiments involving this species.

Supplementary Table S1. Larval mortality following exposure to TMP and DMSO

A. TMP dissolved in DMSO only or containing 10% FBS

			TMP			TMP+10%FBS		
**Time (hrs)**		CTRL	0.1mM	1mM	10mM	0.1mM	1mM	10mM
**Dead**	24hs	0	0	24	44	0	39	49
	48hs	0	65	39	19	65	24	14
**Alive**	48 hs	65 (100%)	0 (0%)	0(0%)	0(0%)	0(0%)	0(0%)	0(0%)
**Total Larvae**		65	63	63	65	65	63	63

B. DMSO toxicity

**Time (hrs)**		**CTRL**	**0.1% DMSO**	**1% DMSO**	**10% DMSO**
**Dead**	24hs	0	1	65	65
	48hs	0	21	0	0
	72hs	0	43	0	0
**Alive**	72hs	65 (100%)	0(0%)	0(0%)	0(0%)
**Total Larvae**		65	65	65	65
